# Renal Endometriosis Mimicking Cystic Renal Tumor: Case Report and Literature Review

**DOI:** 10.3389/fmed.2021.684474

**Published:** 2021-06-21

**Authors:** Ye Yang, Xinxin Zhao, Ying Huang

**Affiliations:** ^1^Department of Ultrasound, Sheng Jing Hospital of China Medical University, Shenyang, China; ^2^Department of Hospice, Sheng Jing Hospital of China Medical University, Shenyang, China

**Keywords:** endometriosis, renal endometriosis, computed tomography, cystic renal tumor, ultrasonography

## Abstract

**Background:** Endometriosis mainly affects female pelvic tissues and organs, and the presence of endometriosis in the kidney is extremely rare.

**Case Presentation:** We report a case of a 48-year-old woman who presented with intermittent hematuria. She was found to have a cystic mass on renal ultrasonography, and contrast-enhanced computed tomography (CT) showed slight enhancement of the cystic wall and septa. These findings were indicative of cystic renal tumor. The patient subsequently underwent partial right nephrectomy. Histopathology revealed endometriosis of the right renal parenchyma. The patient recovered well and had no evidence of a recurrent renal mass at the 3 months' follow up.

**Conclusion:** The possibility of renal endometriosis should be considered in a female patient with a cystic renal mass and clinical symptoms related to the menstrual cycle.

## Introduction

Endometriosis is a common disease occurring in women of reproductive age. Most lesions occur in the reproductive system (1), and endometriosis of the kidneys is rare (2). Because of the insufficient knowledge of renal endometriosis, it can be easily misdiagnosed. Herein, we report our experience with renal endometriosis with the goal of increasing the scholarly awareness of this rare entity.

## Case Presentation

A 48-year-old married woman presented to our hospital with intermittent gross hematuria for 3 months. She had regular menstrual cycle of 28 days without pain around her waist line or abdominal pain and no history of gynecologic surgery. She denied a family history of cancer, endometriosis, genetic and psychosocial diseases.

**Figure 1:**
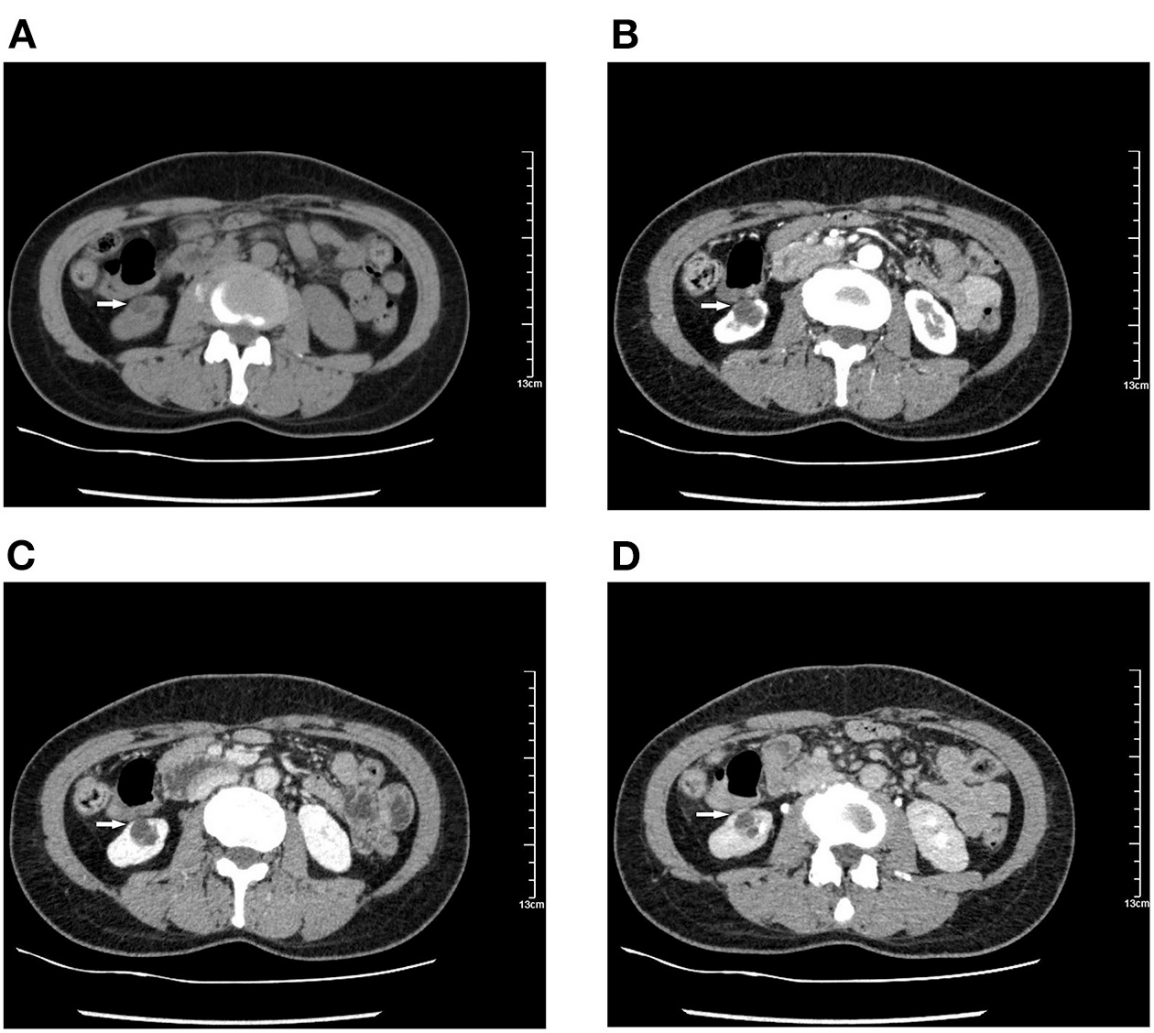
Computed tomography (CT) shows a hypodense mass of 1.8 × 1.5 × 1.4 cm in the lower pole of the right kidney. Contrast-enhanced CT scan shows a polycystic mass with slight enhancement of the wall and septa and no enhancement in the cystic part. The right lower pole of the kidney is partially depressed, and contrast-enhanced images show local thinning of the renal cortex during cortical enhancement. **(A)** Unenhanced images. **(B–D)** With contrast. **(B)** Renal cortical phase. **(C)** Renal corticomedullary phase. **(D)** Renal excretory phase.

**Figure 2:**
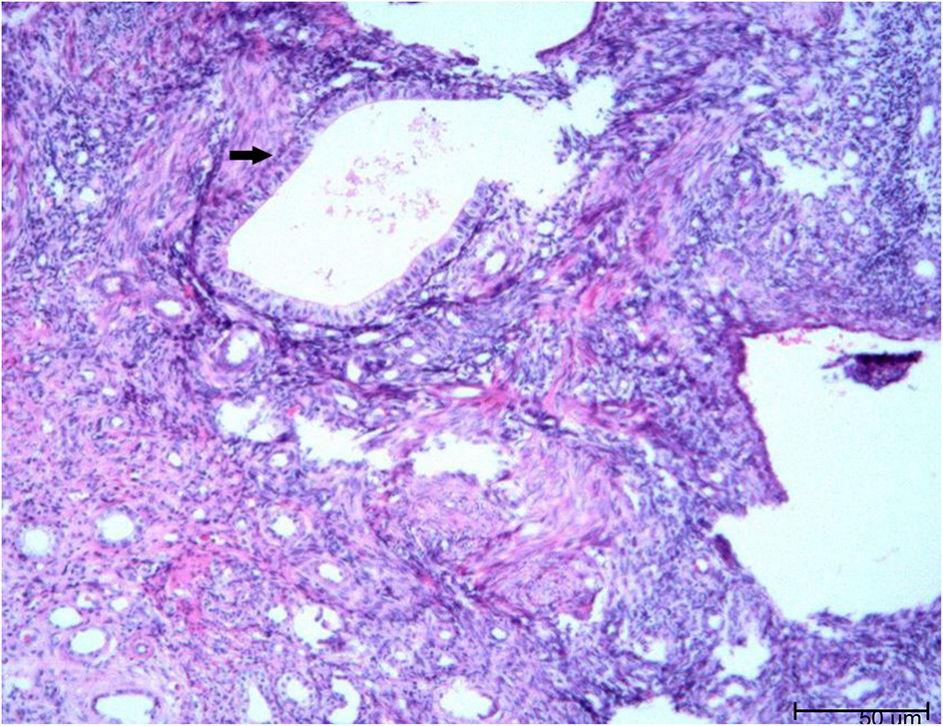
The microscopic pathology proved the diagnosis of renal endometriosis characterized by endometrial glands and embedded stromal cells [hematoxylin and eosin stain (H and E), × 200 magnification].

There was no obvious mass on the palpation of the bilateral kidney area, and no percussion pain. An ultrasound of the urinary system was recommended first. Conventional ultrasound examination was performed under standardized settings using Toshiba ultrasound systems (Aplio500, Tokyo, Japan) (ultrasound transducer: PVT-375BT, frequency 3.5 MHz). Ultrasonography showed a cystic mass in the lower pole of the right kidney with septa and well-circumscribed. The thickness of the cystic mass wall is about 4 mm. Doppler color flow imaging showed a small signal in the wall of the mass, but no blood flow was detected in the cystic part. Contrast-enhanced computed tomography (CT) of the kidney was then performed. It showed a heterogeneous hypodense mass in the lower pole of the right kidney, measuring ~1.8 × 1.5 × 1.4 cm (Figure 1). The mass was polycystic, with slight enhancement of the wall and septa and no enhancement of the cystic area. Contrast filling of the mass was not observed during the excretory phase. The right lower pole of the kidney was partially depressed, and there was some local thinning of the renal cortex. Given the slight enhancement of the mass, the possibility of cystic renal tumor could not be ruled out, and surgical resection was planned. Laparoscopic right renal mass resection was subsequently performed at our hospital on the 7th day after her menstruation. After successful anesthesia, the surgeon opened the lateral retroperitoneum under laparoscopy, pushed the colon away and opened the Gerota's fascia, then carefully dissociated the right kidney. The right lower pole of the kidney was partially irregular and contracted. A well-demarcated cystic tumor with the size of 2.5 × 2.0 × 2.0 cm could be seen at the lower pole of the right kidney after surgical incision, which involved the renal parenchyma but had clear boundaries, and the mass was easily peeled off. The mass composed of brown central area showed several branching cavities, with 5 mm wide homogenous light-yellow zone at its periphery. Perioperatively, the tumor was confined to the right kidney without extracapsular invasion, which did not encroach on the perirenal fat sac or surrounding organs. The mass was completely resected, with an ~0.5 cm margin of normal renal tissue. The resected mass had a diameter of ~1.5 cm. There were several capsular spaces containing brown fluid, and the cut surface of the mass was yellowish. Microscopic examination confirmed the diagnosis of renal endometriosis characterized by endometrial glands and embedded stromal cells (Figure 2). Immunohistochemical analysis revealed that the stromal cells and epithelial cells were positive for estrogen receptor (ER), progestin receptor (PR), and vimentin, which further supported the diagnosis of renal endometriosis. Histopathology was reviewed by two senior pathologists separately, without any atypia. The patient recovered well after the surgery, and there were no obvious space-occupying lesions by ultrasonography in the kidney at the 3 months follow-up. The patient is very satisfied with the treatment of this disease.

## Discussion

Endometriosis is a common gynecologic disease, occurring in up to 10% of women, mainly of reproductive age (1). The main pathologic changes are cyclic shedding of the ectopic endometrium and surrounding tissue fibrosis with the formation of heterotopic nodules. Endometriosis usually affects pelvic tissues and organs (3), but extragenital endometriosis can occur anywhere in the body (4–40), as shown in Table 1. Urinary tract involvement is uncommon and primarily manifests in the bladder, followed by the ureters and kidneys, at a ratio of 40:5:1 (41). Marshall first reported renal endometriosis in 1943 (20), and only 16 pathologically confirmed cases have been reported in the past 30 years (Table 2) (42–56). The median age was 37 years (range, 23–53 years).

**Table 1:** Extragenital endometriosis.

**References**	**Lesion location**	**Size (cm)**	**Age (years)**	**Symptom**	**Endometrial history**	**Preoperative diagnosis**	**Diagnosis method**	**Treatment**	**Outcome**
**Digestive system**									
Fluegen et al. (4)	Liver	12 × 9.5	32	Right upper quadrant abdominal tenderness	No	Hepatic cyst	Histopathology	Pericystectomy	Not mentioned
Saldaña et al. (5)	Gallbladder	3.5 × 2.5	26	Right upper quadrant abdominal pain	Not mentioned	Intramural lesions	Histopathology	Cholecystectomy	Not mentioned
Monrad-Hansen et al. (6)	Pancreas	Not mentioned	43	Acute epigastric pain	Yes	Endometriosis	Histopathology	Laparoscope	Recovery
Anaf et al. (7)	Stomach	6	28	Abdominal pain	Not mentioned	Endometriosis	Histopathology	Laparotomy	Recovery
Buldanl et al. (8)	Colon	3.5	40	Abdominal pain	Not mentioned	Not mentioned	Histopathology	Surgery	Recovery
Choi and Yunaev (9)	Appendix	Not mentioned	29	Abdominal pain	No	Distal small bowel obstruction	Histopathology	Appendicectomy	Recovery
Pramateftakis et al. (10)	Sigmoid	Not mentioned	47	Acute onset of pain in the left lower abdomen	Not mentioned	Rectosigmoid mass	Histopathology	Surgery	Recovery
Shi and Fan (11)	Rectum	0.8 × 0.6	37	Anal mass prolapse accompanied by bloody stools	Not mentioned	Rectum mass	Histopathology	Surgery	Not mentioned
Kołodziejczak et al. (12)	Anus	3.5 × 2.1	34	Painful perianal mass	Not mentioned	Anal abscess	Histopathology	Surgery	Recovery
**Peritoneum**									
Wang et al. (13 )	Peritoneum	1.86	24	Rapidly enlarging abdominal	Not mentioned	Massive hemorrhagic ascites	Histopathology	GnRHa	Improvement
Sima et al. (14)	Mesentery	9	29	Infertility	Not mentioned	Cystic mass	Histopathology	Laparoscopy	Recovery
Athwal et al. (15)	Lesser omentum	Not mentioned	37	Crampy abdominal pain	Yes	Not mentioned	Histopathology	No	Not mentioned
**Retroperitoneal**									
Lu et al. (16)	Retroperitoneal	3.7 × 3.6	30	Intermittent upper abdominal pain	Not mentioned	Retroperitoneal cystic lesion	Histopathology	Surgery	Not mentioned
**Respiratory system**									
Mignemi et al. (17)	Nasal mucosa	Not mentioned	35	Epistaxis and nasal pain	Yes	Mucosal lesions	Histopathology	GnRHa	Improvement
Tong et al. (18)	Pulmonary lobe	Not mentioned	29	Catamenial hemoptysis	No	Pulmonary endometriosis	Histopathology	Thoracoscopic resection	Recovery
Nizami et al. (19)	Pleura	Not mentioned	32	Catamenial hemothorax	Not mentioned	Not mentioned	Histopathology	Parietal pleurectomy	Recovery
**Urinary system**									
Marshall (20)	Kidney	9	40	Swelling and pain in the left flank	Not mentioned	Left kidney mass	Histopathology	Surgery	Recovery
Rajaian et al. (21)	Ureter	0.7	26	Left lower quadrant abdominal pain	No	Polypoidal mass	Histopathology	Surgery	Recovery
Alsinan et al. (22)	Bladder	2.8 × 2 × 3.1	25	Intermittent right flank pain	Yes	Bladder mass	Histopathology	Surgery, GnRHa	Recovery
**Circulatory system**									
Ceccaroni et al. (23)	Pericardium	Not mentioned	28	Abdominal pain with irradiation up to the right shoulder	Yes	Pericardium mass	Histopathology	Surgery	Recovery
**Nervous system**									
Maniglio et al. (24)	Cerebrum	Not mentioned	39	Catamenial epilepsy with hallucinations	Yes	Cerebral hemosiderosis deposits in the globus pallidus	Images and treatment responses	GnRHa	Remission of the disease
Motamedi et al. (25)	Lumbosacral plexus	4.5	37	Pelvic pain	Yes	Lesion involving the Sciatic nerve	Images and treatment responses	Hormone therapy with Decapeptide	Pain subsided completely
Steinberg et al. (26)	Conus medullaris	2.5 × 1	29	Progressive difficulty voiding and lower-extremity radiating radicular pain		Conus medullaris lesion	Histopathology	Surgery	Not mentioned
Teixeira et al. (27)	Sciatic nerve	Not mentioned	26	Pain and weakness in the lower left limb	Not mentioned	Left sciatic nerve cystic lesion	Histopathology	Microsurgery	Not mentioned
**Bone**									
Dongxu et al. (28 )	Spinal vertebrae	Not mentioned	33	Lower back pain	Not mentioned	Vertebral body tumor-like lesion	Histopathology	Surgery	Recovery
**Articulation**									
Jelsma et al. (29 )	Knee joint	Not mentioned	17	Left knee pain and swelling	Not mentioned	Thickened synovium	Images and treatment responses	Hormonal treatment	symptoms completely resolved
**Muscle**									
Akhtar Haseeb (30 )	Rectus sheath	4.1 × 2.8	47	Lower abdominal pain	No	Rectus sheath lesion	Histopathology	Surgery	Recovery
Zhao et al. (31)	Psoas muscle	12	28	Lower left abdominal and back pain	Not mentioned	Psoas abscess	Histopathology	GnRHa, surgery	Alleviation of symptoms
Niro et al. (32)	Iliopsoas	4.2 × 2.9	29	Chronic pelvic pain with dysmenorrhea	No	Iliopsoas endometriotic lesion	Histopathology	GnRHa, surgery	Recovery
Ferhatoglu and Senol (33)	External oblique muscle fascia	4 × 3	29	Left lower-quadrant pain	Not mentioned	Superficial soft tissue mass	Histopathology	Surgery	Recovery
Anderson et al. (34)	Diaphragm	not mentioned	38	Cyclic shoulder pain	No	Diaphragm lesion	Histopathology	Surgery	Recovery
**Visual organ**									
Sharghi et al. (35)	Eyelid	0.1 × 0.2	41	A growth on her right upper eyelid	Not mentioned	Non-blanching macule of telangiectasias	Histopathology	Surgery	Follow-up
**Other locations**									
Fernández Vozmediano et al. (36 )	Surgical scar	Not mentioned	32	A tumor of 3 years' evolution on the abdomen	Not mentioned	Abdomen lesion	Histopathology	Surgery	Not mentioned
Dashraath et al. (37)	Umbilicus	2.5 × 2.0 × 2.0	30	Enlarging umbilical nodule	Not mentioned	Umbilical nodule	Histopathology	Surgery, GnRHa	Not mentioned
Fong et al. (38)	Inguinal	3	41	Right groin swelling	No	Inguinal hernia and abscess	Histopathology	Surgery	Not mentioned
Jiménez et al. (39)	Round ligament	3	35	Constant mass grew and became painful	No	Inguinal endometriosis	Fine-needle aspiration cytology	Surgery	Recovery
Schuster and Mackeen (40)	Fetal	6.5 × 4.8.5.2	18	A large fetal pelvic mass at 35 weeks' gestation	Not mentioned	Fetal pelvic mass	Histopathology	Surgery	Recovery

*GnRHa, gonadotrophin releasing hormone agonist*.

**Table 2:** Case reports of renal endometriosis.

**References**	**Age (years)**	**Kidney involvement**	**Endometrial history**	**Number of lesions**	**Maximum size (cm)**	**Symptom**	**Preoperative diagnosis**	**Diagnosis method**	**Treatment**	**Outcome**
Hajdu and Koss (42)	49	Left kidney	No	1	1.5 × 1.0	Low back pain and gross hematuria	No	Microscopic examination	No	Die
Gauperaa and Stalsberg (43)	23	Right kidney	Not mentioned	1	2	Tender to palpation in the region of the right kidney	No	Histopathology	Nephrectomy	Recovery
Bazaz-Malik et al. (44)	40	Upper pole of the right kidney	Not mentioned	2	2	Dull aching pain in the right loin	Hydatid cyst	Histopathology	Nephrectomy	Not mentioned
Hellberg et al. (45)	25	Upper pole of the left kidney	Endometriotic cyst of left ovary	1	4	Macroscopic haematuria and back-pain	Cystic tumour	Ultrasound-guided FNA	GnRHa	Effective
Benchekroun et al. (46)	35	Left kidney	Not mentioned	1	Not mentioned	Fever and pyuria	Pyelonephritis	Histopathology	Nephrectomy	Not mentioned
Gupta et al. (47)	40	Right kidney	Endometriotic cyst of right ovary	Multiple	2	Pain in the lower abdomen	No	Ultrasound-guided FNA	Not mentioned	Not mentioned
Dutta et al. (48)	38	Right kidney	Endometriotic cyst of left ovary	Multiple	1	Abdominal pain	No	FNA	GnRHa	The lesions regressed slowly
Yaqub et al. (49)	25	Left kidney	Not mentioned	1	25 × 15	Lower abdominal pain	Mesenteric cyst, hydronephrotic kidney or a retroperitoneal tumour	Histopathology	Nephrectomy	Recovery
Dirim et al. (50)	46	Lower pole of the left kidney	No	1	15 × 9.7 × 9.5	Left lumbar pain and lumbar mass	Subcapsular hematoma	Histopathology	The renal capsule was incised and excised after hematoma drainage	Recovery
Jiang et al. (51)	42	Middle and lower of the right kidney	No	1	13.5 × 12 × 12	Right flank pain and hematuria	Angiomyolipoma	Histopathology	Nephrectomy, GnRHa	Recovery
Yang et al. (52)	37	Lower pole of the right kidney	Not mentioned	1	7.5 × 5 × 3.5	Dull pain in the right lower back	Renal tumor	Histopathology	Nephrectomy	Not mentioned
Cheng et al. (53)	53	Right kidney	Not mentioned	Multiple	Not mentioned	Intermittent recurrent right flank pain	Right kidney abscess	Histopathology	Nephrectomy after drainage	Recovery
Giambelluca et al. (54)	40	Upper and lower poles of the left kidney	Ovarian endometriosis	Multiple	1.8	No	Hemorrhagic cysts	Histopathology	No	No clinical changes
Giambelluca et al. (54)	39	Mid pole of the left kidney	Ovarian endometriosis	Multiple	<1.0	No	Haemorrhagic cysts	Histopathology	No	No clinical changes
Badri et al. (55)	45	Upper pole of the left kidney	No	1	2.8 × 2.6 × 1.7	Flank pain and intermittent gross hematuria	Renal mass	Histopathology	Partial nephrectomy	Not mentioned
Umair et al. (56)	30	Right kidney	Not mentioned	1	6.5 × 5.9 × 5.7	Dull pain in the right lower back	Renal tumor	Histopathology	Nephrectomy	Not mentioned

*GnRHa, gonadotrophin releasing hormone agonist; FNA, fine-needle aspiration*.

The mechanism of extragenital endometriosis remains controversial. The main theories can be categorized as migratory, embryonic, and immunologic, these theories have been discussed in previous literature (43, 44, 46, 48, 50–54, 56, 57). Migratory theories propose that retrograde menstruation, lymphovascular metastasis, and direct extension allow the endothelial cells to transplant into ectopic sites (46, 48, 50–54, 56, 57). Embryonic theories suggest that endometriosis results from metaplastic changes of Wolffian, Mullerian, and occasionally peritoneal (celomic) structures (43, 44, 46, 48, 50, 51, 53, 54). Immunologic theories suggest that a suboptimal immune response may result in ectopic endometrial implantation (46, 50–54). In these literatures, Bazaz-Malik et al., Dutta et al., and Cheng et al. believe that the pathogenesis of their cases of renal endometriosis is rationally explained by embryonic theory (44, 48, 53).

The qualitative features of Bosniak III cystic masses: cystic masses with one or more thick or irregular enhancing walls or septa without nodular enhancement. The Bosniak Classification, version 2019 defines “thick” as 4 mm or thicker (58). In our case, the thickness of the cystic mass wall is 4 mm and with enhancement. It was classified as Bosniak III according to Bosniak Classification of Cystic Renal Masses, Version 2019 (58). Approximately 50% of Bosniak III masses are malignant (59). The imaging features of multilocular cystic renal neoplasm of low malignant potential are different from benign renal cystic masses. In general, necrosis of carcinoma tends to be central, with a thickened solid peripheral “rind” and a central ill-defined area of non-enhancement (60). Different imaging modalities may have some roles in the diagnosis of renal endometriosis. Shedding of the ectopic endometrium may result in a heterogeneous mass density on CT scan (52). The hyperplastic fibromuscular tissue surrounding the lesion is unevenly enhanced and may show protrusion into the central part (49). The continuous proliferation of muscle fibers around the lesion can distort the surrounding renal parenchyma and change the shape of the kidney. Unlike the CT scan, MRI can show renal pelvis compression or displacement. Mixed cystic-solid masses with fibromuscular hyperplasia and residual blood will produce heterogeneous MRI signals (49). The location, size, and composition (solid vs. cystic) of a renal mass can also be visualized using ultrasound. Ultrasound color Doppler flow imaging can demonstrate blood flow signals in the wall and septa. Ultrasound can also be used to facilitate tissue diagnosis. Ultrasound-guided fine needle aspiration (FNA) biopsy is an accurate and minimally invasive method that can greatly help in the diagnosis of endometriosis. To date, three cases of renal endometriosis have been confirmed by FNA (45, 47, 48). Ultrasound is very useful for detecting endometriosis in the uterus and ovary (61), and most importantly, it is cheap, especially suitable for developing countries.

Pain and hematuria are often intermittent and associated with the menstrual cycle in patients with renal endometriosis. Our patient had no abdominal pain, but her hematuria was cyclical and corresponded to her menstrual cycle. This important information was overlooked at the time of her initial presentation.

The histopathology of renal endometriosis is characterized by endometrial glands and stromal cells involving renal cortex and medulla (62). Immunohistochemical analysis shows stromal cell and epithelial cell positivity for CD10, ER, and PR (62). In our case, the stromal cells and epithelial cells were positive for ER, PR, and vimentin, which further supported the diagnosis of renal endometriosis.

As there are no treatment guidelines for renal endometriosis because of its rarity (51), the treatment should be based on the patient's clinical symptoms, characteristics of the lesion, and the patient's reproductive plans (41). Although renal endometriosis is a benign lesion, surgical treatment is usually considered due to its invasiveness (63). The feasibility of laparoscopic management is now widely proven and may reduce the length of hospital stay (64). Asymptomatic patients with multiple small lesions unchanged during subsequent imaging examinations generally do not require any definitive therapy for renal lesions (54). Hormone therapy, such as GnRH agonists and oral contraceptives, can be used for symptom management (45, 48, 51). Hormonal treatment reduces the pain in the short-term follow up and is the best treatment for patients of reproductive age (64).

## Conclusion

We report a patient who presented with a single small cystic kidney mass and was found to have endometriosis. Although imaging can be helpful, the final diagnosis of endometriosis relies on histopathologic findings. The possibility of renal endometriosis should be considered in a female patient with a cystic renal mass and clinical symptoms related to the menstrual cycle.

## Data Availability Statement

The original contributions presented in the study are included in the article/supplementary material, further inquiries can be directed to the corresponding author.

## Ethics Statement

Written informed consent was obtained from the participant for the publication of this case report. Ethical approval was given by the Medical Ethics Committee of our hospital.

## Author Contributions

YY and YH diagnosed the patient. All authors wrote and revised the manuscript.

## Conflict of Interest

The authors declare that the research was conducted in the absence of any commercial or financial relationships that could be construed as a potential conflict of interest.
